# Phonon-Mediated Quasiparticle Lifetime Renormalizations in Few-Layer Hexagonal Boron Nitride

**DOI:** 10.1021/acs.nanolett.3c02086

**Published:** 2023-08-10

**Authors:** Håkon I. Røst, Simon P. Cooil, Anna Cecilie Åsland, Jinbang Hu, Ayaz Ali, Takashi Taniguchi, Kenji Watanabe, Branson D. Belle, Bodil Holst, Jerzy T. Sadowski, Federico Mazzola, Justin W. Wells

**Affiliations:** † Department of Physics and Technology, University of Bergen, Allégaten 55, 5007 Bergen, Norway; ‡ Department of Physics, Norwegian University of Science and Technology (NTNU), NO-7491 Trondheim, Norway; § Department of Physics and Centre for Materials Science and Nanotechnology, University of Oslo (UiO), Oslo 0318, Norway; ∥ Department of Smart Sensor Systems, SINTEF DIGITAL, Oslo 0373, Norway; ⊥ Department of Electronic Engineering, Faculty of Engineering & Technology, University of Sindh, Jamshoro 76080, Pakistan; # International Center for Materials Nanoarchitectonics, National Institute for Materials Science, 1-1 Namiki, Tsukuba 305-0044, Japan; ∇ Research Center for Functional Materials, National Institute for Materials Science, 1-1 Namiki, Tsukuba 305-0044, Japan; ○ Center for Functional Nanomaterials, Brookhaven National Laboratory, Upton, New York 11973, United States; △ Department of Molecular Sciences and Nanosystems, Ca’ Foscari University of Venice, 30172 Venice, Italy; ▲ Istituto Officina dei Materiali, Consiglio Nazionale delle Ricerche, Trieste I-34149, Italy

**Keywords:** hexagonal boron nitride, graphene heterostructures, many-body interactions, electron−phonon coupling, ARPES

## Abstract

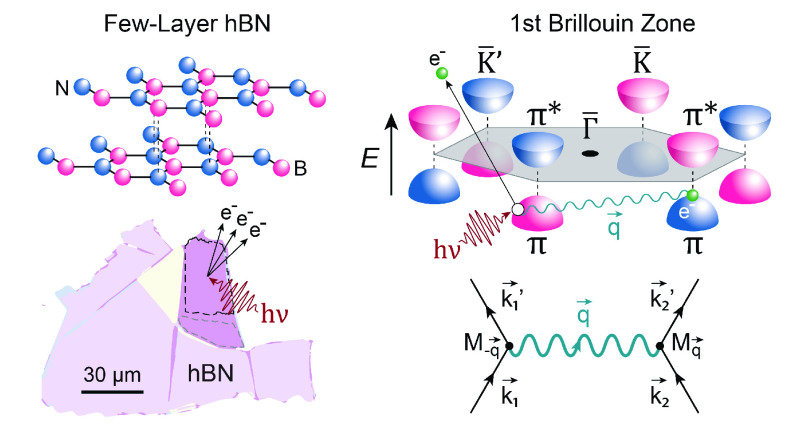

Understanding the collective behavior of the quasiparticles in solid-state systems underpins the field of nonvolatile electronics, including the opportunity to control many-body effects for well-desired physical phenomena and their applications. Hexagonal boron nitride (hBN) is a wide-energy-bandgap semiconductor, showing immense potential as a platform for low-dimensional device heterostructures. It is an inert dielectric used for gated devices, having a negligible orbital hybridization when placed in contact with other systems. Despite its inertness, we discover a large electron mass enhancement in few-layer hBN affecting the lifetime of the π-band states. We show that the renormalization is phonon-mediated and consistent with both single- and multiple-phonon scattering events. Our findings thus unveil a so-far unknown many-body state in a wide-bandgap insulator, having important implications for devices using hBN as one of their building blocks.

Hexagonal boron nitride (hBN) is an inert layered compound that has gained significant attention for its compatibility with the vast majority of low-dimensional van der Waals (vdW) materials.^1−8^ It is strikingly similar to graphene in lateral size, crystalline structure, and Debye frequency, but due to its dissimilar sublattices, it hosts a wide energy band gap separating the valence and conduction bands.^9,10^ For the engineering of vdW heterostructures embedded in the form of devices, hBN has proven to be a key building block due to its large capacitive coupling and current tunneling barrier.^7,11−14^ Furthermore, its chemical inertness, large energy bandgap, and high phonon energies have made it one of the most common dielectrics for use in state-of-the-art, low-dimensional devices that require atomic-scale flatness and negligible interface doping and scattering.^1,3,15−17^ Recently, hBN has been predicted to host a strong electron–phonon coupling which can compromise the performance of hBN-derived electronic devices.^18,19^ However, experimental proof of such couplings has so far been lacking. Herein, we investigate the many-body effects of few-layer hBN supported on graphene. We discover and quantify the predicted electron–phonon coupling and also an additional scattering effect that, together, significantly renormalizes the hBN π-band states. The latter effect is found to be consistent with a scattering process involving multiple phonons. Thus, our findings are crucial for understanding the many-body states in hBN-based devices and for achieving increased control over their performances.

Few (5–10)-layer hBN flakes were exfoliated from bulk material and transferred via a polymer stack onto a substrate of epitaxial graphene on 6H-SiC(0001) (Figure 1a). The material was then heated in ultrahigh vacuum (pressure ≤1 × 10^–9^ mbar) to remove any residues of polymer from the transfer process (see the Supporting Information for details). Using photoemission electron microscopy (PEEM), we selected a high-quality hBN flake of approximately 35 × 20 μm^2^ lateral size (Figure 1b). The crystalline quality across the flake was ascertained from small-area low-energy electron diffraction (μ-LEED, 1.5 μm diameter spot), showing no appreciable variation across the full flake area (Figure 1c). The diffraction pattern taken at an electron energy of 35 eV revealed six first-order spots, as expected for a stack of rotationally aligned hBN layers.

**Figure 1:**
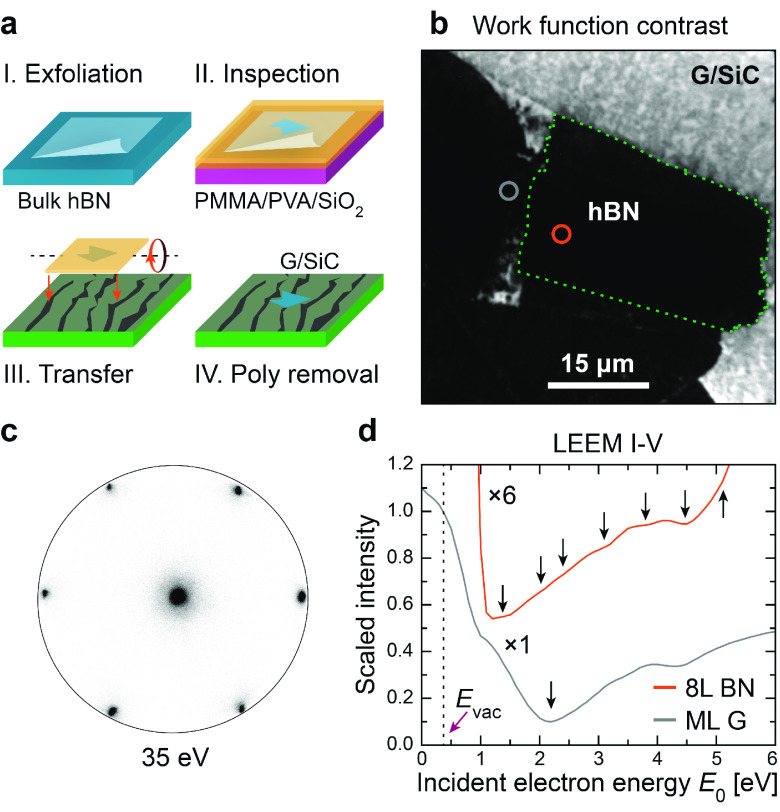
Exfoliated hBN on graphene/SiC. (a) Preparation of few-layer hBN by exfoliating from a bulk crystal and transferring onto graphene/SiC using a polymer (PMMA) film. (b) PEEM micrograph of hBN on graphene/SiC. The region encompassed by the dashed line (green) is the relevant hBN region used for further analysis. (c) μ-LEED from a 1.5 μm diameter area on the hBN region marked in (b), collected with an electron energy of 35 eV. (d) LEEM I-V spectra collected from the circular areas marked in (b). The dips in the I-V curves reveal that the hBN (orange) consists of 8 layers, while the substrate (gray) is mainly monolayer graphene. The curves have been normalized to the maximum intensity at the mirror electron microscopy energy threshold (∼0.4 eV). The hBN curve has been scaled (×6) and offset in intensity for improved readability.

The number of vdW-bonded hBN layers in the flake was ascertained from low-energy electron reflectivity (LEER) measurements (Figure 1d). The incident, coherent electron beam was tuned in the range 0–10 eV, and the corresponding electron reflectivity at each energy was recorded from micrographs of the surface. The averaged I-V characteristics from areas on and adjacent to the hBN flake (circles in Figure 1b) reveal dips in the reflected intensity at energies beyond the mirror electron microscopy energy threshold at ∼0.4 eV. These dips represent the transmission of electrons into discrete and unoccupied states above the vacuum energy level. In the case of graphene or hBN, the minima are given by the unoccupied π*-states of each atomic layer. The number of minima *n* observed can hence be linked directly to the *n* + 1 stacked layers present.^20−23^ From the substrate region (gray), one dominant dip (∼2.1 eV) can be observed between the higher intensity regions (0.5 and 6.0 eV), suggesting the substrate is mainly monolayer graphene on top of -reconstructed SiC(0001).^20^ In comparison, the hBN flake (orange) shows 7 dips originating from 8 stacked layers.^22^ For the electronic structure measurements, we will refer to this 8-layer region from now on.

The electronic structure of 8-layer hBN on graphene is mapped out at room temperature in Figure 2 for binding energies near the top of the π-band (*E*_VBM_). The occupied band structure as shown was measured as a function of constant energy simultaneously across all momentum vectors of the first Brillouin zone (BZ), using an aberration-corrected momentum microscope with its energy and momentum resolutions set to 50 meV and 0.02 Å^–1^, respectively.^24,25^ The measured π-states reveal several signatures of electron scattering. Notably, a broadening appears along the  direction at approximately *E*_VBM_ + 1 eV (blue arrows) where also two faint and linearly dispersing features appear (gray arrow/circles), intersecting with the hBN π-band. Additional strong signs of broadening (green arrows) can be seen along the  direction for energies closer to the valence band maximum (VBM).

Sudden broadenings of the electronic structure like the ones mentioned are typical hallmarks of electron–boson interactions.^26−29^ They signify a direct change in the quasiparticle lifetime τ—or said differently, a reduction of the time an electron uses to fill a photohole.^27^ This phenomenon has already been thoroughly studied in hBN’s sister compound graphene.^30,31^ Therein, spectroscopic signatures of lifetime renormalizations at large binding energies away from the Fermi level (*E*_F_) have been reported.^31^ Their origin has been debated,^32^ but the evidence is pointing toward a strong electron–phonon coupling (EPC) in the σ-bands, mediated bythe sudden onset of electron density of states (eDOS) at their band maxima.^33,34^ The EPC renormalizes the σ-bands with a large mass enhancement (i.e., with a mass-enhancement parameter λ ≈ 1), manifesting itself as energy broadenings and “kinks”.^33^ Additional broadenings near the σ- and π-band extremum points from phonon-mediated intraband (π → π) and interband (σ → π) scattering have also been reported.^34^

To ascertain the origins of the renormalizations observed from the hBN π-band, their magnitudes and binding energies were studied in more detail. In Figure 2c, the measured  wedge of the BZ is plotted along with the energy band half-line widths (∝ τ^–1^) along these high-symmetry directions. The maximum broadening along  appears at ∼175 meV lower binding energy than the π-band vH singularity at M̅. Interestingly, this energy separation matches roughly with the expected scattering energy ℏω_D_^BN^ of the longitudinal optical phonon modes of hBN.^19,35^ This is illustrated in Figure 2c by the semitransparent, blue interaction region overlaid on the measured π-band structure in this energy range. Given its demonstrated occurrence in graphene,^34^ we postulate that the described renormalization comes from intraband π_BN_–π_BN_ electron–phonon scatterings originating at the vH singularity (M̅) of hBN.

**Figure 2:**
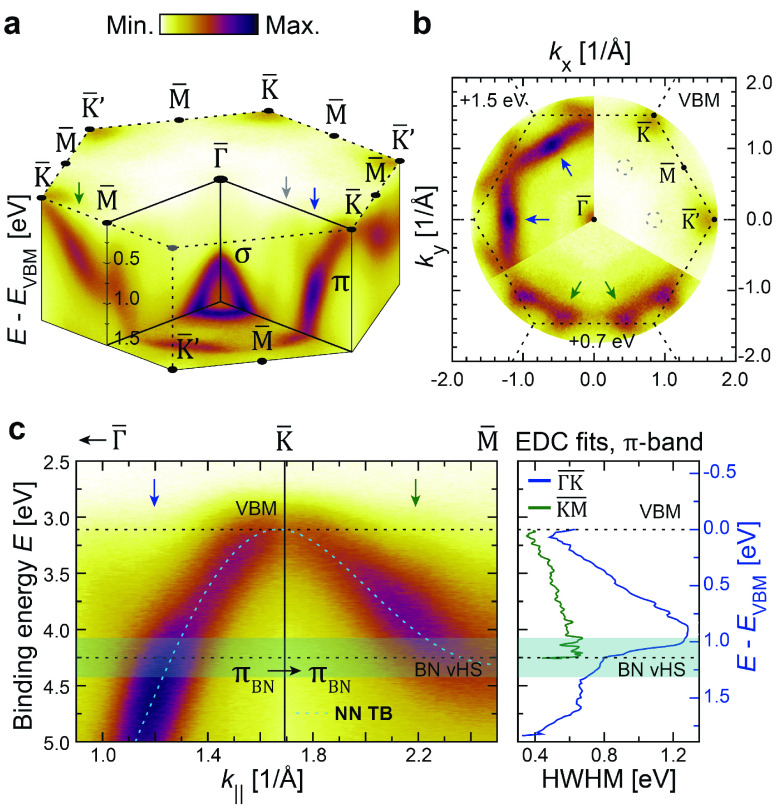
Electronic structure of hBN on graphene/SiC. (a) Volumetric 3D rendering of the hBN electronic structure within the first BZ. A binding energy range of 0–1.5 eV is shown relative to the maximum of the valence (π) band. Prominent scattering interactions are marked by arrows. (b) Sequence of constant energy (*k*_*x*_ vs *k*_*y*_) surfaces at binding energies relative to the valence band maximum (VBM). Each surface is shown as a 1/3 slice of a full pie chart. The scattering interactions from (a) are marked (arrows and circles). (c) Valence band (π) dispersion extracted along the  high-symmetry directions, together with the extracted half-line width (in eV). The ARPES data have been overlaid with a simple nearest-neighbor tight binding calculation (dashed blue). Also marked are the measured energies for the VBM and the π-band van Hove (vH) singularity of hBN. The semitransparent (blue) rectangle marks an interaction region of Δ*E* = 2ℏω_D_^BN^ around the vH energy.

A more rigorous analysis of the observed energy renormalization is partially prevented by the faint linear artifacts that intersect with the hBN π-band at a similar binding energy (blue arrows). Still, their effect is very local in the energy and momentum phase diagram. Naively, these artifacts can be mistaken for the π-band of the underlying graphene layer. However, the graphene π-band is only nearly linear close to the K̅ point, which is found at a radius of approximately 1.7 Å^–1^ relative to Γ̅ in both graphene and hBN.^36,37^ Selective area photoemission measurements of the hBN flake and the adjacent graphene region showed that the two materials were more or less aligned rotationally on top of each other (within 5°). Given the small misalignment, the linear features were situated at the wrong distance from Γ̅ and thus could not be a part of the graphene π-band itself.^38,39^

Additionally, the true graphene π-band should cross the Fermi level.^30^ However, a curvature analysis^40^ of the  wedge from Figure 2c revealed that the anomalous features were visible exclusively at binding energies larger than *E*_VBM_, i.e., where the hBN has a finite eDOS (details in the Supporting Information). This signifies that the observed anomalies involve hBN-dependent transitions that are inelastic in energy and/or momentum. Based on similar observations in few-layer graphene, we infer that they are signatures of secondary electrons ejected from the limited number of unoccupied final states available.^41−43^ Alternatively, these could be from Umklapp-scattered electrons ejected from the underlying graphene, interacting with the hBN as they pass through the flaked material on their way out into vacuum.^44^

Similar to the case of the graphene σ-band, the strong eDOS increase set naturally by the hBN π-band maximum at the K̅ point is expected to enable phonon-mediated energy renormalizations.^19,31,33^ This is also corroborated by our complex self-energy Σ analysis: along the  direction an abrupt increase in half-line width near the VBM can be observed, being a typical hallmark of EPC.^26−28^ At these energies the gradient of the π-band dispersion is small, and its measured energy half-line width translates to the imaginary part of the self-energy (Im Σ) directly.^27,29,45^ The real part of the self-energy (Re Σ) can be found from the discrepancy between the measured band position and the theoretically expected, noninteracting band structure: Re Σ ≡ *E*(**k**) - ε(**k**).^26^

The noninteracting electronic band ε(**k**) can be obtained using various approximations. For example, the π-band of mono- or multilayer hBN can be described by a tight-binding (TB) approximation, which is subsequently fitted to experimental results or first-principles calculations to obtain reasonable hopping parameters.^36,38,46^ However, the resultant ε(**k**) can only be used as an approximation at best, as the fitting will ignore all finite Re Σ contributions from any many-body interactions. Alternatively, the full band structure of the hBN-on-graphene system could be calculated from first-principles, but this requires detailed knowledge of the hBN–substrate interaction and the stacking sequence and rotation of the hBN layers, which goes well beyond the scope of this work.^47,48^

In general, discrepancies between the measured and calculated electronic structures will occur, and their magnitude will depend on the type and level of approximation used. A solution that allows us to circumvent this problem and properly establish the noninteracting “bare” energy band ε(**k**) is to make no rigorous assumptions about its energy dispersion. Instead, the fact that Re Σ and Im Σ are causally related can be exploited so that one is determined from the other via a Kramers–Kronig (K-K) transformation. This methodology has been previously adopted for graphene^31,33^ and is discussed in detail in refs (49 and 50). A first guess for ε(**k**) was found from a nearest-neighbor TB calculation using the parameters *t*_1_ = 2.92 eV and Δ_BN_ = 4.3 eV.^9,51^ Then, keeping the energy and momentum of the VBM fixed, the shape of ε(**k**) was adjusted to satisfy the causality between the Σ components via the K-K transformation. The resulting, noninteracting π-band and Σ components are shown in Figure 3.

**Figure 3:**
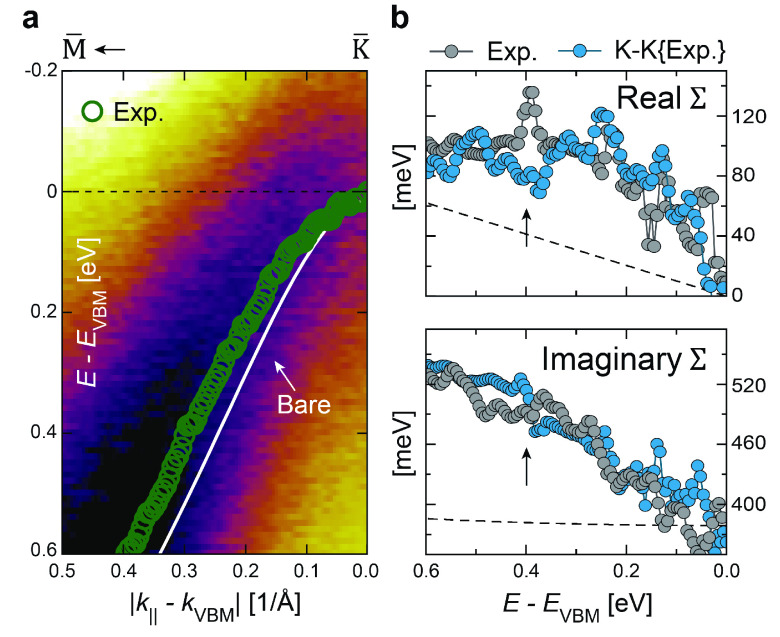
Energy renormalization near the hBN π-band maximum. (a) Measured electronic structure of the hBN π-band, overlaid with the renormalized band position (green dots) as determined by curve fitting and the unrenormalized band (white line) from a Kramers–Kronig analysis. (b) Self-consistent Re Σ and Im Σ of the π-band corresponding to the spectrum shown in (a). Both experimental Σ contributions (gray) have been overlaid by a Kramers–Kronig transformed equivalent (blue), estimated from the opposite experimental component. An abrupt energy renormalization (arrows) on a background of electron–impurity and electron–electron interactions (dashed lines) can be distinguished from both Σ components.

At a glance, a prominent energy renormalization can be readily distinguished from both the Re Σ and Im Σ near the VBM (arrows in Figure 3b). Its characteristic functional shape and weakly increasing background toward larger binding energies indicate the presence of both electron–phonon and electron–electron interactions.^26,28^ Indeed, had this feature originated from a crossing between hBN and the anomalous band features near the vH energy, it would have existed exclusively as a local broadening across the intersectional region.^34,44^ Instead, the observed energy renormalization persists at larger binding energies as expected.^27^

A closer inspection of the measured Σ components revealed a fine structure of multiple distinct spectral signatures at well-defined binding energies. Hence, Σ_ph_ due to EPC was estimated from the data by subtracting the electron–impurity and electron–electron scattering contributions as shown in Figure 3b (details in the Supporting Information). The resulting Re Σ_ph_, with its leading features labeled, is shown in Figure 4a (blue dots). We note that the renormalized and unrenormalized bands could not be unambiguously distinguished from one another at energies smaller than the instrument resolution (50 meV). Hence the first few data points of Re Σ_ph_ have been omitted.^52^

From the resultant Re Σ_ph_, several distinct and peak features can be readily distinguished. When referenced to the VBM, the first two appear at energies similar to the in-plane acoustic phonons of few-layer hBN.^35^ A minor feature can be resolved at approximately the hBN Debye energy ℏω_D_^BN^ (dashed line).^19^ Surprisingly, three more features can also be seen at energies larger than ℏω_D_^BN^, the first feature at approximately *E*_VBM_ +250 meV.

To better understand the observed peaks and quantify their individual contributions to the overall EPC, the Eliashberg function α^2^*F*(ω) and corresponding electron mass-enhancement factors λ_*n*_ were estimated. The former approximates the DOS of the interacting phonon modes present, and the latter quantifies the strengths of their coupling to the electrons.^27^ Using a maximum entropy method (MEM) procedure,^52−54^ α^2^*F*(ω) was extracted from the data and the cumulative mass enhancement from the different phonon modes estimated as . The results, along with the corresponding best fit to Re Σ_ph_, are shown in Figure 4. The energies and λ_*n*_ values of the electron–phonon interactions are summarized in Table 1.

Immediately, the two lowest energy features in α^2^*F*(ω) can be correlated to peaks 1 and 2 as observed from Re Σ_ph_. Both signify a strong EPC in the acoustic energy regime, having mass-enhancement factors of λ_1_ = 0.39 and λ_2_ = 0.21, respectively. Such strong coupling to the acoustic modes has already been predicted theoretically in hBN systems thicker than 1 ML.^18^ Also, the observed, dominant feature (3) above the Debye energy is faithfully reasserted with λ_3_ = 0.19. The smaller features (i–iii), although visible from the measured Re Σ_ph_, were not unambiguously resolved by the MEM analysis when accounting for the thermal broadening of the measurements.

Based on their energies, the observed peaks above the Debye energy cannot be explained by single-phonon scattering alone. Their presence, however, hints at the possibility of multiphonon scattering events. Consecutive electron scattering by multiple phonons has been predicted from theory, suggesting that substantial scattering rates may occur in insulating and semiconducting polar materials.^56,57^ Additionally, experimental signatures of multiphonon scattering in few-layer hBN have been suggested from inelastic electron tunneling spectroscopy (IETS) measurements.^35^ However, a mode-specific quantification of any multiple-phonon interactions with electrons has, until now, not been presented. From the known phonon energies of hBN^35,55^ we are able to suggest different two-phonon combinations to explain the observed higher-order peaks above the Debye energy. These, along with suitable phonon modes for the EPCs at lower energies, are also summarized in Table 1.

**Figure 4:**
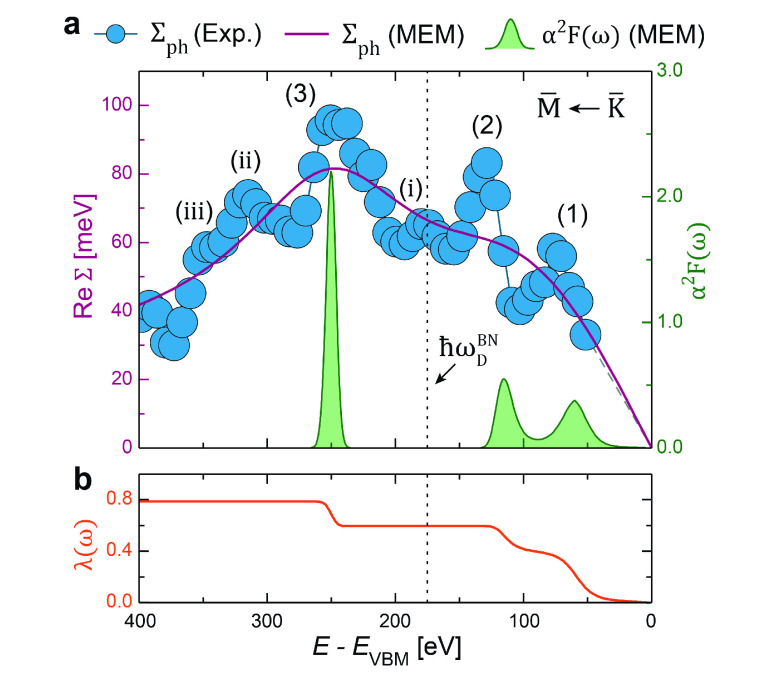
Signatures of electron–phonon interactions near the hBN π-band maximum. (a) Experimentally determined, real self-energy contribution from electron–phonon interactions Re Σ_ph_ (in blue), overlaid with a best-fit model (purple line) and its corresponding Eliashberg function α^2^*F*(ω) (in green) as estimated by MEM analysis. An experimental error of σ = 5 meV for each data point was estimated from the measured noise level and is shown by the size of the data point spheres. Additionally, the hBN Debye energy ℏω_D_^BN^ ≈ 175 meV has been indicated relative to the VBM (dashed vertical line). (b) Integrated mass-enhancement factor λ(ω) of the electron–phonon coupling estimated from α^2^*F*(ω) shown in (a).

**Table 1:** Summary of the Peak Assignments from Figure 4, along with Their Energies, Contributions to λ, and Suggested Scattering Phonons^a^

peak	*ℏω* (meV)	λ_*n*_	phonon mode(s)
1	60 ± 12	0.39	TA_BN_/ZO_BN_
2	115 ± 9	0.21	TA_BN_/LA_BN_/ZO_BN_
(*i*)	175 ± 16	minor	TO_BN_/ LO_BN_
3	245 ± 5	0.19	2 × LA_BN_
(*ii*)	315 ± 16	minor	2 × TO_BN_
(*iii*)	345 ± 13	minor	2 × LO_BN_

a The entries in parentheses are distinguishable from Re Σ but were not resolved by the MEM analysis. The phonon mode suggestions have been based on their measured and calculated energy values from Refs (35 and 55).

While electron–phonon scattering between the hBN and the underlying graphene cannot be ruled out completely, we can render it unlikely in the present case. Previous ARPES studies of graphene-on-hBN have indicated that coupling of the two materials should lead to large (i.e., Fröhlich) polaron formation at the interface.^58^ In contrast, no such signatures of large polaron formation can be observed here.^59^ Examining other exfoliated hBN flakes with different thicknesses and rotational alignment with the substrate led to the same conclusion. Alternatively, one could suggest that the EPC peaks presented in Table 1 resulted from electron–phonon scattering between the hBN and graphene bands.^34^ However, intermaterial EPC for the current system is expected to be weak when compared to the theoretical EPC of few-layer hBN^18^ and the mass-enhancement demonstrated from our analysis.

We conclude that the apparent correspondence between the measured EPCs and scattering with graphene is coincidental. We reiterate, however, that our MEM analysis was unable to properly distinguish all of the engaging hBN phonon modes. This was in part caused by the achievable instrumental resolution but primarily by the thermal broadening at room temperature. For instance, further investigating the potential coupling to the lowest-energy modes using a more sensitive method, e.g. helium atom scattering,^60,61^ could provide additional insight. However, this would require larger-area single-crystalline samples to be achieved.

In summary, we have reported the existence of several EPC processes that renormalize the π-band electronic structure of hBN. Together, they cause an increase in the electron scattering rate and a decrease in the energy state lifetime. This may have severe implications for the electron transport in any hBN-adjacent conducting layers, e.g. in vdW heterostructures. Thus, our work not only sheds light on the complex many-body ground state of few-layer hBN but also provides valuable insight into the possible scattering mechanisms that may hamper the performances of hBN-based electronic devices.

## Supporting Information Available

Further details on the hBN sample preparation, the measurement parameters, the curvature analysis of the hBN π-band, the estimation of the “bare” energy band dispersion ε(**k**), and the interpretation of the measured self-energies (PDF)
