# Supplementary material for: Phonon-Mediated Quasiparticle Lifetime Renormalizations in Few-Layer Hexagonal Boron Nitride
Source: Nano Lett. 2023 Aug 10;23(16):7539–45. doi: 10.1021/acs.nanolett.3c02086 (PMC10450811; doi:10.1021/acs.nanolett.3c02086)
Supplement: Supplementary file 1 — nl3c02086_si_001.pdf [file nl3c02086_si_001.pdf]

Supporting Information:
Phonon-Mediated Quasiparticle Lifetime Renormalizations in Few-Layer Hexagonal Boron Nitride

Håkon I. Røst,[1,2,*] Simon P. Cooil,[3] Anna Cecilie Åsland,[2] Jinbang Hu,[2] Ayaz Ali,[4,5] Takashi Taniguchi,[6] Kenji Watanabe,[7] Branson D. Belle,[4] Bodil Holst,[1] Jerzy T. Sadowski,[8] Federico Mazzola,[9,10] and Justin W. Wells[2,3,†]

[1]*Department of Physics and Technology, University of Bergen, Allégaten 55, 5007 Bergen, Norway.*

[2]*Department of Physics, Norwegian University of Science and Technology (NTNU), NO-7491 Trondheim, Norway.*

[3]*Department of Physics and Centre for Materials Science and Nanotechnology, University of Oslo (UiO), Oslo 0318, Norway.*

[4]*Department of Smart Sensor Systems, SINTEF DIGITAL, Oslo, 0373, Norway.*

[5]*Department of Electronic Engineering, Faculty of Engineering & Technology, University of Sindh, Jamshoro, 76080, Pakistan.*

[6]*International Center for Materials Nanoarchitectonics, National Institute for Materials Science, 1-1 Namiki, Tsukuba 305-0044, Japan.*

[7]*Research Center for Functional Materials, National Institute for Materials Science, 1-1 Namiki, Tsukuba 305-0044, Japan.*

[8]*Center for Functional Nanomaterials, Brookhaven National Laboratory, Upton, New York, 11973, USA.*

[9]*Department of Molecular Sciences and Nanosystems, Ca' Foscari University of Venice, 30172 Venice, Italy.*

[10]*Istituto Officina dei Materiali, Consiglio Nazionale delle Ricerche, Trieste I-34149, Italy.*

S1. SAMPLE PREPARATION

Crystals of bulk hexagonal boron nitride (hBN) were prepared according to a 'temperature-gradient method' at high pressure and temperature, using barium boron nitride ($Ba_3B_2N_4$) as a solvent system [1]. Thin flakes of hBN were micromechanically exfoliated [2] from the bulk crystals and transferred onto a PMMA/PVA/SiO_2 stack. Suitable samples of few-layer hBN on the polymer stack were identified by optical contrast microscopy (Fig. S1**a**), and subsequently transferred onto substrates of monolayer graphene on 6H-SiC(0001) [3], using a dry transfer method at 80 °C [4]. Excess polymer from the transfer was removed by annealing the hBN/graphene/SiC heterostructures under ultrahigh vacuum (pressure $\leq 1 \times 10^{-9}$ mbar) to 300 °C for a few hours, followed by several flashes to 600 °C for short durations.

* hakon.rost@uib.no

† j.w.wells@fys.uio.no

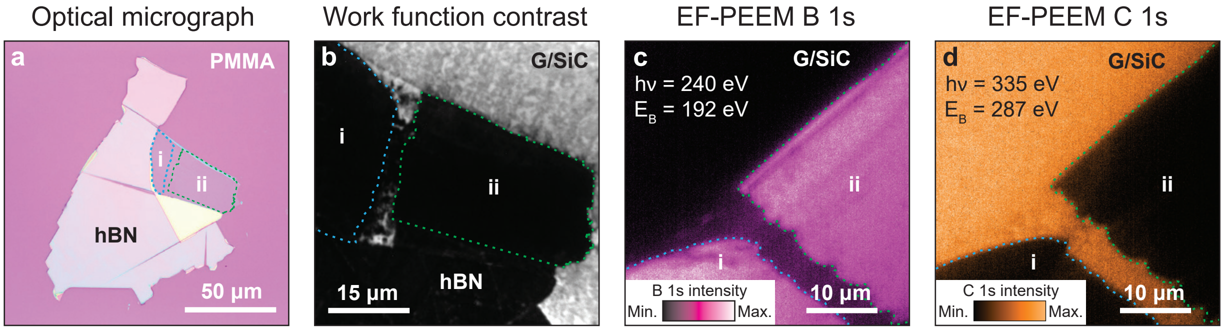

FIG. S1. Preparation and characterization of a few-layer hBN sample. **a**: Optical micrograph of the exfoliated hBN flake on PMMA that was selected for further transfer onto graphene on SiC. **b**: The same exfoliated flake measured after the transfer onto graphene on SiC, using work function contrast PEEM near the secondary electron cutoff ($h\nu \approx 5.2$ eV). The regions i and ii encompassed by the dashed, blue and green lines in both subfigures were the ones used for concomitant LEEM and PEEM studies. **c**, **d**: Energy-filtered PEEM micrographs of the exfoliated hBN flake, performed at the binding energies E_B of the B 1s and C 1s core levels. The B 1s signal predominantly occurs within regions i and ii, whereas the C 1s signal can only be observed from the graphene substrate.

S2. LEEM AND PEEM MEASUREMENTS

LEEM, μ-LEED, and XPEEM measurements were performed at the ESM 21-ID-2 (LEEM/PEEM) beamline of National Synchrotron Light Source II (NSLS-II), using an Elmitec aberration-corrected LEEM/PEEM. The presence and position of a suitable and properly grounded exfoliated hBN flake on the graphene substrate were ascertained by LEEM and spatially resolved, surface-sensitive XPEEM measurements of the B 1s and C 1s core levels (Figs. S1**c** and S1**d**, inelastic mean-free path < 0.5 nm). Additionally, dispersive plane XPS measurements of the B 1s, C 1s, and O 1s were measured within the region of the flake to ascertain the composition of the sample. The low-energy electron reflectivity (LEER) imaging measurements (discussed in the main text) were performed in the range 0-10 eV, using an incident and coherent electron beam. The μ-LEED was collected using a 1.5 µm diameter selective area aperture placed within the region of the hBN flake.

Further photoemission measurements in both real and k-space were performed using an aberration-corrected, energy-filtered photoemission electron microscope (EF-PEEM) working at an extraction voltage 12 kV (NanoESCA III, Scienta Omicron GmbH). Real-space imaging was performed with a pass energy $E_\mathrm{P} = 50$ eV, using a 150 µm contrast aperture, a 0.5 mm entrance slit, and a non-monocromated Hg source ($h\nu \approx 5.2$ eV) for photoexcitation. With these settings, the microscope had the nominal real space and energy resolutions $\Delta x = 35$ nm and $\Delta E = 100$ meV,

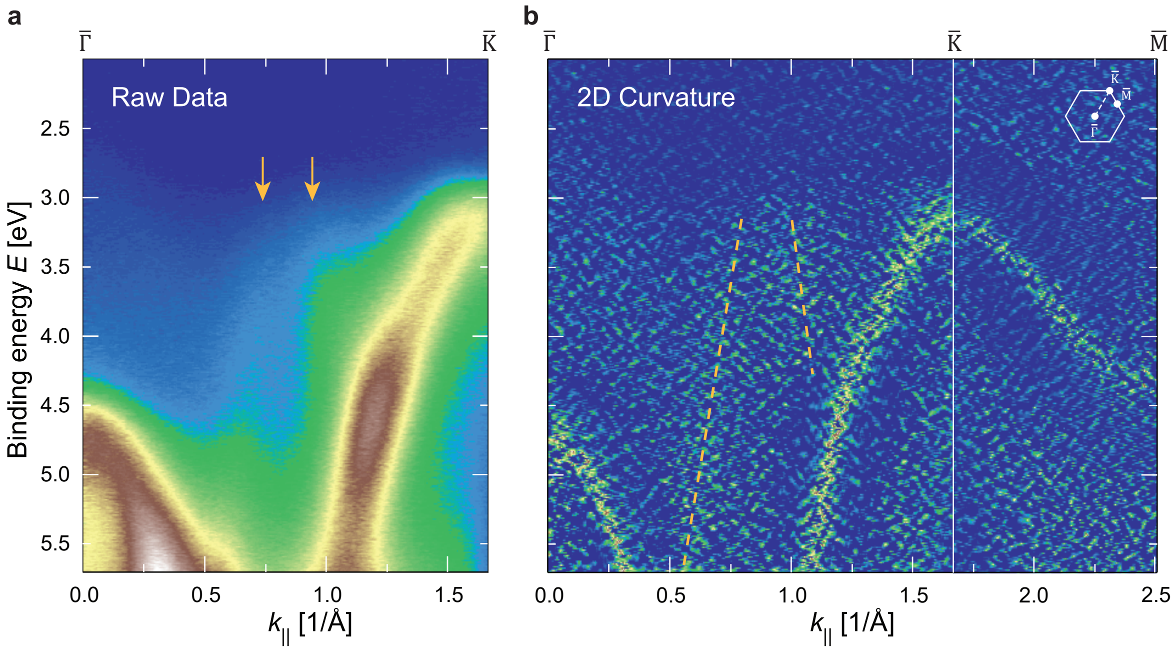

FIG. S2. Curvature analysis of the $\bar{\Gamma}-\bar{\mathrm{K}}-\bar{\mathrm{M}}$ wedge. The positions of the anomalous, near-linearly dispersing bands have been highlighted by arrows (**a**) and dashed lines (**b**). From the two-dimensional curvature plot (**b**), the anomalous bands diminish in intensity at binding energies above the hBN valence band maximum.

respectively. The hBN bandstructure was reconstructed from a series of measured constant energy surfaces (CES, E vs. k_x, k_y) obtained in the diffractive plane mode of the same momentum microscope [5, 6]. The bandstructure measurements were restricted to an area within the hBN flake by introducing an iris aperture of $\approx 30 \times 15\,\mu\mathrm{m}^2$. Using a He I photoexcitation source ($h\nu = 21.22\,\mathrm{eV}$), CES were acquired at $E_\mathrm{P} = 25\,\mathrm{eV}$ with a 0.5 mm entrance slit to the energy filter, yielding energy and momentum resolutions of $\Delta E = 50\,\mathrm{meV}$ and $\Delta k = 0.02\ \mathrm{\AA}^{-1}$, respectively. Each CES was acquired 10 meV apart and with a k-space field of view of $4.3\ \mathrm{\AA}^{-1}$, i.e. spanning beyond the 1st Brillouin zone of hBN. All measurements were performed at room temperature ($\approx 300\,\mathrm{K}$).

S3. CURVATURE ANALYSIS OF $\bar{\Gamma} - \bar{\mathbf{K}} - \bar{\mathbf{M}}$

To assess the presence, shape, and intensity of the anomalous features observed from the hBN ARPES measurements, a two-dimensional (2D) curvature analysis of the $\bar{\Gamma} - \bar{\mathrm{K}} - \bar{\mathrm{M}}$ wedge was performed (see Ref. 7 for details). The raw data and its resulting 2D curvature plot are shown in Fig. S2. The near-linearly dispersing anomalous features can be distinguished near halfway along $\bar{\Gamma} - \bar{\mathrm{K}}$, as indicated by arrows in Fig. S2**a**. Notably, their intensity diminishes at binding energies above the hBN valence band maximum as shown in Fig. S2**b**. Also, no energy-shifted replicas of the hBN π-band from polaron formation can be distinguished from the 2D curvature analysis [8, 9].

S4. ROTATIONAL AVERAGING OF THE ELECTRONIC STRUCTURE

Before extracting anything from the measured bandstructure, the three-dimensional data cone (k_x, k_y, E) was replicated $n = 6$ times. Each copy was rotated around the energy axis, i.e. the $\bar{\Gamma}$ point of the BZ, by $\varphi = n(2\pi/6)$ before adding them all together. This was done to minimize any geometrically induced differences in the photoionization matrix elements [10], and furthermore to improve the signal-to-noise ratio of the measured bands.

S5. APPROXIMATING THE NON-INTERACTING π-BAND

Energy distribution curves (EDCs) of the π-band were fitted over the relevant energy range using a Lorentzian line shape, approximating the background by an error function to accommodate the visible 'step' in intensity near the VBM. The band's peak position and linewidth (in eV) were extracted and used to estimate the $\mathrm{Re}\,\Sigma$ and $\mathrm{Im}\,\Sigma$, respectively, using an analysis procedure similar to the ones demonstrated in Refs. 11–14. As an initial guess for the unperturbed π-band, a nearest-neighbor tight-binding (TB) calculation was performed with $t_1 = 2.92\,\mathrm{eV}$ and $\Delta_{\mathrm{BN}} = 4.3\,\mathrm{eV}$ [15, 16]. The initial bare band curve obtained from the TB calculation was approximated by a fifth-degree polynomial over the same energy range as the fitted experimental band. Finally, its shape was iteratively adjusted to achieve consistency between the measured, real and imaginary parts of the self-energy (Σ_{exp}) through the Kramers-Kronig transform [11, 12].

S6. ESTIMATING THE INELASTIC BACKGROUND OF $\mathrm{Re}\,\Sigma$ AND $\mathrm{Im}\,\Sigma$

To estimate the measured inelastic energy losses due to non-bosonic interactions, a simplistic model Σ was constructed and fitted to the measured Σ_{exp}. The model consisted of a linear combination of contributions describing the electron-impurity ($\Sigma_{\text{el-imp}}$), electron-electron ($\Sigma_{\text{el-el}}$), and electron-phonon ($\Sigma_{\text{el-ph}}$) scattering. The free parameters of the model were in turn adjusted to fit the measured $\mathrm{Im}\,\Sigma_{\mathrm{exp}}$ by minimizing their root mean square (RMS) difference, and subsequently, Kramers-Kronig transformed to find the model $\mathrm{Re}\,\Sigma$. Each fitting parameter uncertainty was estimated from a relative RMS increase of $\pm 5\%$.

The term $\mathrm{Im}\,\Sigma_{\text{el-imp}}$ was modeled as a constant broadening of 380 meV ($\propto T$), estimated from the full width at half maximum (FWHM) of the fitted EDC at the $\bar{\mathrm{K}}$ point. We note here that using the FWHM directly will overestimate $\mathrm{Im}\,\Sigma_{\mathrm{imp}}$, as it also contains additional, constant energy broadening from the instrument's finite energy resolution. The term $\mathrm{Im}\,\Sigma_{\text{el-el}}$ was modeled as a

logarithmically corrected, quadratic expression for a two-dimensional Fermi liquid [17, 18]:

$$\beta \cdot [E - E_{\mathrm{VBM}}]^2 \ln |E - E_{\mathrm{VBM}}|, \tag{S1}$$

with $\beta = 3.0 \pm 0.3\ \mathrm{eV}^{-1}$.

Finally, $\mathrm{Im}\,\Sigma_{\text{el-ph}}$ was modeled by a sum of contributions $\mathrm{Im}\,\Sigma^{(i)}_{\text{el-ph}}$ $(i = 1, 2)$ from two separate phonon modes with different maximum energies. The density of states $F^{(i)}(\omega)$ of each phonon mode was described using a two-dimensional and isotropic Debye model ($\propto \omega$), having a distinct and characteristic maximum (cutoff) frequency ω_i. Using Eliashberg formalism, each contribution (i) to $\mathrm{Im}\,\Sigma_{\text{el-ph}}$ from the individual electron-phonon interactions was calculated as [18, 19]:

$$\mathrm{Im}\,\Sigma^{(i)}_{\text{el-ph}}(\omega, T) = \pi \int_0^{\omega_i} \alpha^2 F^{(i)}(\omega') \cdot [1 + 2n(\omega', T) + f(\omega + \omega', T) - f(\omega - \omega', T)]\mathrm{d}\omega', \tag{S2}$$

where $n(\omega, T)$ and $f(\omega, T)$ are the boson and fermion distributions, respectively. The term $\alpha^2 F^{(i)}(\omega) = (\lambda^{(i)}_{\mathrm{ph}}/2)(\omega/\omega_i)$ for $\omega < \omega_i$, where $\lambda^{(i)}_{\mathrm{ph}}$ is the dimensionless mass enhancement factor for coupling to the phonon mode i. Above each cut-off frequency ω_i, the corresponding $\alpha^2 F^{(i)}(\omega) = 0$. The optimized two-phonon Eliashberg model yielded peaks at energies $\hbar\omega_1 = 185 \pm 35$ meV and $\hbar\omega_2 = 335 \pm 15$ meV below the VBM, with the mass-enhancement factors $\lambda_1 = 0.18 \pm 0.04$ and $\lambda_2 = 0.36 \pm 0.03$, respectively.

S7. ESTIMATING THE ELECTRON-PHONON COUPLING FROM $\mathrm{Re}\,\Sigma$

The real counterpart to the $\mathrm{Im}\,\Sigma_{\text{el-ph}}$ expression in Eq. S2 is defined as [20]:

$$\mathrm{Re}\,\Sigma_{\text{el-ph}}(\omega, T) = \int_{-\infty}^{\infty} \mathrm{d}\nu \int_0^{\omega} \alpha^2 F(\omega') \cdot \frac{2\omega'}{\nu^2 - \omega'^2} \cdot f(\nu + \omega, T)\mathrm{d}\omega'. \tag{S3}$$

Here, $\alpha^2 F(\omega)$ is the total phonon density of states (DOS) weighted by the effective strength α^2 of the electron-phonon coupling. Thus, it describes the phonon modes participating in the interaction and contributing to $\mathrm{Re}\,\Sigma_{\text{el-ph}}$. From $\alpha^2 F(\omega)$, the total mass-enhancement factor λ of the electrons can be found by [21]:

$$\lambda_{\mathrm{ph}} = 2 \int_0^{\omega_{\max}} \frac{\alpha^2 F(\omega')}{\omega'} \mathrm{d}\omega', \tag{S4}$$

where $\omega_{\max}$ is the maximum observable phonon energy. If $\mathrm{Re}\,\Sigma_{\text{el-ph}}$ is known, e.g., from ARPES measurements, then $\alpha^2 F(\omega)$ can be estimated from the expression in Eq. S3 by integral inversion. The result can then be used to predict which (combinations of) phonon modes participate, and their interaction strength λ_{ph} with the electrons can be found from Eq. S4.

To overcome the challenge of mathematical instability posed by the direct integral inversion of $\mathrm{Re}\,\Sigma_{\text{el-ph}}$ a maximum entropy method (MEM) analysis procedure was employed. A detailed outline

of the method and its theoretical foundations can be found in Refs. 22–24. Therefore, only a brief summary is given here. The optimum approximation to the experimental $\mathrm{Re}\,\Sigma_{\text{el-ph}}$ is obtained by minimizing the functional:

$$L = \frac{\chi^2}{2} - aS, \tag{S5}$$

where χ^2 is the standard deviation between the measured and calculated $\mathrm{Re}\,\Sigma_{\text{el-ph}}$. The term aS serves to prevent overfitting of the experimental data by imposing physical constraints on how the $\alpha^2F(\omega)$ term should look. S is the Shannon-Jaynes entropy [23]:

$$S = \int_0^\infty \mathrm{d}\omega' \left[\alpha^2F(\omega') - m(\omega') - \alpha^2F(\omega') \ln \frac{\alpha^2F(\omega')}{m(\omega')}\right], \tag{S6}$$

with the constraint model $m(\omega)$ reflecting our *a priori* knowledge of $\alpha^2F(\omega)$. The term a is a coefficient in the conditional probability of $\alpha^2F(\omega)$ under the constraints set by $m(\omega)$ [25]. In 'classical' MEM, the probability defined by a, S and $m(\omega)$ is maximized to minimize L [22].

For the analysis of the hBN π-band, the constraint model was chosen to be:

$$m(\omega) = \begin{cases} m_0(\omega/\omega_\mathrm{A})^2, & \text{if } 0 \le \omega < \omega_\mathrm{A}, \\ m_0, & \omega_\mathrm{A} \le \omega \le \omega_\mathrm{max}, \\ 0, & \text{otherwise.} \end{cases} \tag{S7}$$

This model reflects the expected quadratic increase in DOS up to the maximum energy $\hbar\omega_\mathrm{A}$ of the first distinguishable acoustic mode [26]. The constant m_0 is positive, thus making $m(\omega)$ positive definite over the relevant energy range. The energy $\hbar\omega_\mathrm{max}$ defines the cutoff energy for the observed electron-phonon interactions.

Initially, m_0 was set to approximately the average value expected for $\alpha^2F(\omega)$. The energy parameter $\hbar\omega_\mathrm{A}$ was set to approximately the $E - E_\mathrm{VBM}$ value where the first peak in the experimental $\mathrm{Re}\,\Sigma_\mathrm{ph}$ could be observed (see Fig. 4**a** in the main text). Finally, $\hbar\omega_\mathrm{max}$ was fixed at $E - E_\mathrm{VBM} = 400$ meV at the step-like cut-off in the experimental $\mathrm{Re}\,\Sigma_\mathrm{ph}$ and $\mathrm{Im}\,\Sigma_\mathrm{ph}$ (Fig. 3**b**, main text). In minimizing L, the parameters m_0 and $\hbar\omega_\mathrm{A}$ were iteratively adjusted, achieving the final values $\hbar\omega_\mathrm{A} = 60$ meV and $m_0 = 0.35$. This resulted in a satisfying fit to $\mathrm{Re}\,\Sigma_\mathrm{ph}$, with the constraint function recreating the main feats of $\alpha^2F(\omega)$ (i.e., positive definite, initial quadratic increase). Yet, it was still sufficiently structureless for an unbiased fitting of the data [23].

REFERENCES

[1] K. Watanabe, T. Taniguchi, and H. Kanda, Direct-bandgap properties and evidence for ultraviolet lasing of hexagonal boron nitride single crystal, Nat. Mater. **3**, 404 (2004).

[2] P. Blake, E. W. Hill, A. H. Castro Neto, K. S. Novoselov, D. Jiang, R. Yang, T. J. Booth, and A. K. Geim, Making graphene visible, Appl. Phys. Lett. **91**, 063124 (2007).

[3] X. Z. Yu, C. G. Hwang, C. M. Jozwiak, A. Köhl, A. K. Schmid, and A. Lanzara, New synthesis method for the growth of epitaxial graphene, J. Electron Spectros. Relat. Phenomena **184**, 100 (2011).

[4] C. R. Dean, A. F. Young, I. Meric, C. Lee, L. Wang, S. Sorgenfrei, K. Watanabe, T. Taniguchi, P. Kim, K. L. Shepard, and J. Hone, Boron nitride substrates for high-quality graphene electronics, Nat. Nanotechnol. **5**, 722 (2010).

[5] C. Tusche, A. Krasyuk, and J. Kirschner, Spin resolved bandstructure imaging with a high resolution momentum microscope, Ultramicroscopy **159**, 520 (2015), special Issue: LEEM-PEEM 9.

[6] C. Tusche, Y.-J. Chen, C. M. Schneider, and J. Kirschner, Imaging properties of hemispherical electrostatic energy analyzers for high resolution momentum microscopy, Ultramicroscopy **206**, 112815 (2019).

[7] P. Zhang, P. Richard, T. Qian, Y.-M. Xu, X. Dai, and H. Ding, A precise method for visualizing dispersive features in image plots, Rev. Sci. Instrum. **82**, 043712 (2011).

[8] C. Chen, J. Avila, S. Wang, Y. Wang, M. Mucha-Kruczyński, C. Shen, R. Yang, B. Nosarzewski, T. P. Devereaux, G. Zhang, and M. C. Asensio, Emergence of Interfacial Polarons from Electron–Phonon Coupling in Graphene/h-BN van der Waals Heterostructures, Nano Lett. **18**, 1082 (2018).

[9] C. Franchini, M. Reticcioli, M. Setvin, and U. Diebold, Polarons in Materials, Nat. Rev. Mater. **6**, 560 (2021).

[10] R. P. Day, B. Zwartsenberg, I. S. Elfimov, and A. Damascelli, Computational framework chinook for angle-resolved photoemission spectroscopy, npj Quantum Mater. **4**, 1 (2019).

[11] A. A. Kordyuk, S. V. Borisenko, A. Koitzsch, J. Fink, M. Knupfer, and H. Berger, Bare electron dispersion from experiment: Self-consistent self-energy analysis of photoemission data, Phys. Rev. B **71**, 214513 (2005).

[12] I. Pletikosić, M. Kralj, M. Milun, and P. Pervan, Finding the bare band: Electron coupling to two phonon modes in potassium-doped graphene on Ir(111), Phys. Rev. B **85**, 155447 (2012).

[13] F. Mazzola, T. Frederiksen, T. Balasubramanian, P. Hofmann, B. Hellsing, and J. W. Wells, Strong electron-phonon coupling in the σ band of graphene, Phys. Rev. B **95**, 075430 (2017).

[14] F. Mazzola, C.-M. Yim, V. Sunko, S. Khim, P. Kushwaha, O. J. Clark, L. Bawden, I. Marković, D. Chakraborti, T. K. Kim, M. Hoesch, A. P. Mackenzie, P. Wahl, and P. D. C. King, Tuneable electron–magnon coupling of ferromagnetic surface states in $PdCoO_2$, npj Quantum Mater. **7**, 1 (2022).

[15] J. Robertson, Electronic structure and core exciton of hexagonal boron nitride, Phys. Rev. B **29**, 2131 (1984).

[16] F.-L. Shyu, Electronic and optical properties of boron nitride nanoribbons in electric field by the tight-binding model, Physica B Condens. Matter **452**, 7 (2014).

[17] M. Higashiguchi, K. Shimada, M. Arita, Y. Miura, N. Tobita, X. Cui, Y. Aiura, H. Namatame, and M. Taniguchi, High-resolution photoemission study of electron–electron interaction in the Ni(111) surface state, J. Magn. Magn. Mater. **310**, e743 (2007).

[18] S. Hüfner, *Very High Resolution Photoelectron Spectroscopy*, Vol. 715 (Springer Berlin, Heidelberg, 2007) Chap. 2, pp. 25–32.

[19] B. Hellsing, A. Eiguren, and E. V. Chulkov, Electron-phonon coupling at metal surfaces, J. Phys. Condens. Matter **14**, 5959 (2002).

[20] P. Hofmann, I. Y. Sklyadneva, E. D. L. Rienks, and E. V. Chulkov, Electron–phonon coupling at surfaces and interfaces, New J. Phys. **11**, 125005 (2009).

[21] G. Grimvall, *The Electron-phonon Interaction in Metals* (North-Holland Publishing Company, 1981).

[22] J. E. Gubernatis, M. Jarrell, R. N. Silver, and D. S. Sivia, Quantum monte carlo simulations and maximum entropy: Dynamics from imaginary-time data, Phys. Rev. B **44**, 6011 (1991).

[23] J. Shi, S.-J. Tang, B. Wu, P. T. Sprunger, W. L. Yang, V. Brouet, X. J. Zhou, Z. Hussain, Z.-X. Shen, Z. Zhang, and E. W. Plummer, Direct Extraction of the Eliashberg Function for Electron-Phonon Coupling: A Case Study of Be($10\bar{1}0$), Phys. Rev. Lett. **92**, 186401 (2004).

[24] S.-J. Tang, J. Shi, B. Wu, P. T. Sprunger, W. L. Yang, V. Brouet, X. J. Zhou, Z. Hussain, Z.-X. Shen, Z. Zhang, and E. W. Plummer, A spectroscopic view of electron–phonon coupling at metal surfaces, Phys. Status Solidi B **241**, 2345 (2004).

[25] J. Skilling, *Maximum Entropy and Bayesian Methods*, edited by J. Skilling, Vol. 36 (Springer Dordrecht, 1989) Chap. 3.

[26] C. Kittel, *Introduction to Solid State Physics*, 8th ed. (John Wiley & Sons, Inc., 2021) Chap. 4.
